# Supplementation with Tex261 provides a possible preventive treatment for hypoxic pulmonary artery hypertension

**DOI:** 10.3389/fphar.2022.1028058

**Published:** 2022-11-03

**Authors:** Shaokun Chen, Xiaozhen Wei, Xu Zhang, Mengge Yao, Zhihuang Qiu, Liangwan Chen, Li Zhang

**Affiliations:** ^1^ Department of Cardiac Surgery, Fujian Medical University Union Hospital, Fuzhou, China; ^2^ Department of Pathophysiology, The School of Basic Medical Sciences, The Key Laboratory of Fujian Province Universities on Ion Channel and Signal Transduction in Cardiovascular Diseases, Fuzhou, China; ^3^ Fujian Provincial Key Laboratory of Neurodegenerative Disease and Aging Research, Institute of Neuroscience, School of Medicine, Xiamen University, Xiamen, Fujian, China

**Keywords:** pulmonary artery hypertension, smooth muscle cells, Tex261, Sec23, ndrg1

## Abstract

**Objectives:** Pulmonary artery hypertension (PAH) is a serious disease for which there is no effective treatment. Its pathogenesis is complex and has not yet been clarified. Tex261 is a protein-coding gene whose functional enrichment nodes include the transporter activity of COP II. However, the role of Tex261 in PAH remains unknown.

**Methods:** Sugen5416/Hypoxic PAH models were established, and pulmonary arteries (PAs) were isolated for proteomic sequencing. The binding sites between Hif-1α and Tex261 were verified by dual-luciferase reporter gene assay. Cell proliferation was detected by MTS and EdU assays. For determination of the preventive and therapeutic effects of Tex261, intratracheal instillation of adeno-associated virus (AVV6) with Tex261 vectors was performed.

**Results:** Tex261 was screened according to the proteomic sequencing data. Hif-1α inhibited Tex261 promoter activity under hypoxia. Decreased Tex261 expression promoted PASMC proliferation. Tex261 regulated Sec23 *via* the Ndrg1-mediated Akt pathway. Tex261 overexpression improved the pressure and vessel remodeling of PAs induced by Sugen5416/hypoxia.

**Conclusion:** Hypoxia suppressed Tex261 expression through Hif-1α activation. The decreased Tex261 could promote Ndrg1 and depress Akt activity and then inhibit Sec23 activity, which leads to cell proliferation and vessel remodeling. Elevated Tex261 has some preventive and therapeutic effects on rats with PAH.

## 1 Introduction

Pulmonary artery hypertension (PAH) is diagnosed based on a resting pulmonary artery pressure >25 mmHg. This condition is characterized by pulmonary artery hyperplasia and pulmonary artery remodeling (PAR), leading to right heart failure and even death (Mejía Chew et al., 2016; Simon and Hedderich, 2020). Abnormal proliferation of pulmonary artery smooth muscle cells (PASMCs) is the main cause of pulmonary vascular pressure increases and poor efficacy of drugs (Harding et al., 2003; Kim and George, 2019). Therefore, reversing PASMC proliferation is the key to the treatment of PAH.

Testis expressed 261 (Tex261) is a protein-coding gene whose functional enrichment nodes include coat protein II (COP II) transporter activity. Studies have reported that Tex261 may be involved in regulating excitatory toxic cell death induced by NMDA receptor activation (Miller et al., 2003). Tex261 shares homology with StAR, a steroidogenic acute regulatory protein, but may have different functions (Miller et al., 2003). Therefore, Tex261 is thought to be involved in the formation of COP II, mediating protein transport from the endoplasmic reticulum to the Golgi apparatus (Mossessova et al., 2003). Tex261, as a target gene of miR-28-5p, affects cell proliferation, survival, and apoptosis in PCa. However, the function and possible mechanisms of Tex261 in PAH are unknown.

In this study, significantly different Tex261 expression in Sugen5416/hypoxia induced PAH rats was identified by proteomic sequencing. We investigated the regulatory effect of hypoxia on Tex261 and its role in hypoxia-induced PASMC proliferation and further explored the underlying mechanisms, which will provide a new theoretical basis for the pathogenesis of PAH and identify potential therapeutic and preventive targets for PAH.

## 2 Materials and methods

### 2.1 Establishment of hypoxia models

Male SD rats were supplied by the animal center of Fujian Medical University. We used the animals under the guidelines and principles of the animal experiment committee of Fujian Medical University as well as the experimental program. For animal studies, all *in vivo* procedures were approved by the local authorities (Regierung von Unterfranken) and conformed to the guidelines of the European Parliament directive 2010/63/EU on the protection of animals used for scientific purposes. Rats (200 g) were randomly divided into the control group and hypoxia with Su5416 group (Sugen5416/Hypoxia (Su/Hx)). Su5416 (20 mg/kg, one time) was intraperitoneally injected (IP) before hypoxia. Rats were kept in a hypoxic (FiO_2_ 10%) or normal (FiO_2_ 21%) environment for 3 weeks (W). The right ventricle pressure (PVP) was monitored through the right jugular vein cannula. The rats were killed by cervical dislocation after anesthesia (20% urethane, 5 mg/kg, IP), and then, the heart and lung tissues were collected. The left and right atria and blood vessels were cut off, and the right ventricle (RV) was separated from the left ventricle (LV) and the interventricular septum (S). These samples were dried and weighed separately, and then, the RV/(LV + VS.) was calculated as the right ventricular mass index (RVMI).

### 2.2 Tandem mass tags

The pulmonary arteries (PAs) of rats were quantitatively analyzed by TMT proteomics (LC-BIO Technologies (Hangzhou) Co., Ltd.). Based on criteria for screening differentially expressed proteins (fold-change>1.2, *p* < 0.05), a total of 811 differentially expressed proteins were obtained: 356 were upregulated and 455 were downregulated. The detailed procedures were described in our previous study (Zhang et al., 2019). Heatmaps were obtained based on the heatmap function of R language. Moreover, the heatmaps of the top 20 upregulated and downregulated proteins with the most significant changes are shown (standardized heatmaps of hypoxia/control). In this study, the Tex261 protein (*p* < 0.001) was chosen for functional and mechanistic studies.

### 2.3 Hematoxylin-eosin staining and masson staining

Rats were anesthetized with 20% urethane (5 mg/kg, IP), followed by cervical dislocation. Lung tissues were collected, and the detailed procedures were described previously (Zhang et al., 2014; Zhang et al., 2021). Briefly, tissues were cut into 5 µm thick sections and fixed in 4% formaldehyde for 24 h. After the samples were embedded in paraffin and sectioned, the slides were subjected to HE staining and Masson staining according to the manuals. The morphology and collagen deposition of PAs were observed under a microscope. The vascular media thickness and collagen area were measured by Image-Pro Plus 6.0 software, and then the ratio of the wall thickness of PAs to the artery diameter, and the ratio of the collagen positive staining area (blue) to the total area were calculated. The data were used for statistical analysis.

### 2.4 Immunofluorescence

The sections were placed in 0.1 mol/L citrate repair solution (pH = 6), heated in microwave oven for 6 min at medium heat to slightly boiling, maintained at medium and low heat for 10 min, and cooled naturally for 20–30 min after stopping heating (6 min × 4 times). The sections were blocked with serum for 30 min and then incubated with primary antibodies overnight at 4°C. The next day, the secondary antibodies were reacted for 1 h in the dark. After washing, DAPI was added for 10 min away from light. Finally, the slides were mounted with glycerol, and immediately observed under a fluorescence microscope.

### 2.5 *In situ* hybridization

For inactivation of endogenous enzymes, the slices were dehydrated and reacted with 3% H_2_O_2_ for 10 min, and then, 3% citric acid with freshly diluted pepsin was added and digested at 37°C for 30 min. After that, sections were washed and fixed with 1% paraformaldehyde, and then, 20 µl of prehybridization solution was incubated at 38°C for 3 h. The solution was discarded, and 20 μl of hybridization solution was added to each slice and incubated overnight at 38°C.

The targeted *TEX261* gene sequences were as follows:

5′-ACT​ACC​TTG​CAG​AGC​TGA​TTG​AAG​AGT​ACA​CGG​TGG​CCA​C-3’
5′-TCA​CCA​ACC​TGG​TCT​ACT​TTG​GCC​TTC​TCC​AGA​CCT​TCC-3’
5′-ACT​TCA​CCA​AAG​GCA​AGC​GAG​GCA​AGC​GCT​TAG​GCA​TCC​T-3’

The next day, sections were washed successively twice with 2xSSC, 0.5xSSC, and 0.2xSSC at 37°C for 15 min. The slides were reacted successively with blocking for 30 min, biotinylated mouse anti-digoxigenin for 60 min, strept avidin-biotin complex (SABC) for 20 min, and then biotinylated peroxidase for 20 min, at 37°C. After displaying color visualization with DAB, hematoxylin was counterstained before the sections were observed under a microscope.

### 2.6 Pulmonary artery smooth muscle cell culture

Pulmonary artery smooth muscle cells (PASMCs) were cultured by enzyme digestion (Wang et al., 2019; Zhang et al., 2021). Briefly, lung tissues were removed by thoracotomy after normal rats were anesthetized and then PAs were isolated under a microscope. The PAs were isolated and placed in the enzyme solution to digest for 30–40 min. The cell suspension was placed into a culture flask with 4 ml of DMEM containing 1% penicillin and 10% fetal bovine serum. After cells grew over 90% of the culture flask, they were passaged. Cells passaged 1–5 times were used for experiments. Before hypoxia, the culture medium was replaced with DMEM containing 5% FBS, and then the cells were placed under a 3% O_2_ condition for 24–48 h.

### 2.7 Cell transient transfection

For silencing or overexpression of Tex261, PASMCs were transfected with RNAi or vectors, and the detailed processes are shown in our previous work (Cangul et al., 2002; Zhang et al., 2021).

#### 2.7.1 Transfection of siRNA

Cells in six-well plates were replaced with DMEM before transfection. Lipofectamine RNAiMAX transfection reagent (Invitrogen) and siRNAs (IBSBIO, Shanghai) were diluted with DMEM. Five microliters of transfection reagent with 100 µl of DMEM and 60 pmol of siRNA with 100 µl of DMEM were mixed and placed for 20 min. Then, 200 µl of the mixture was added to each well, mixed gently, and incubated at 37°C for 6 h. Then, the medium was changed, and the cells were cultured for another 24 h. The siRNAs and nontargeted control sequences with FAM tags were as follows:

Tex261-1:5′-GGCUGUCGCUGUUCAUCCAGG-3′
Tex261-2:5′-CCAGCAGAAUCAUCAAAUACA-3′
Tex261-3:5′-GCGAUGACGUGGUCUCCAAUU-3′
Ndrg1-1:5′-CGAAGACCACCCUACUCAAGA–3′
Ndrg1-2:5′-GGUCUGUGAUAGCACGGAAAU–3′
Ndrg1-3:5′-GGAUCUUGGAGUUGCUAGAGG–3′
NC control:5′-UUCUCCGAACGUGUCACGUTT-3′

#### 2.7.2 Plasmid transfection

100 µl strain was added to each tube and shaked overnight. Follow the instructions of the plasmid extraction kit. 10 µl of plasmid was added with endonuclease reaction buffer, restriction nuclease and water, and placed at 37°C for 1–2 h for enzyme digestion. The digestion products were subjected to agarose gel electrophoresis to test whether the digestion was successful. The required fragment was cut down and connected with the carrier. The competent state was made according to the transformation steps, and the above linked products were tested and cultured on coated plates at 37°C for 12–16 h. The monoclones were selected and put into liquid medium containing LB and AMP, and the bacteria were shaken for 4–5 h. The products were detected by electrophoresis. 100 µl of the correct band was extracted, added to LB, AMP + culture medium, shaken overnight, and took 1 ml solution for sequencing. The cloning vector was pcDNA3.1 (+). Cloning sites of Tex261 was BamHI-EcoRI and Sec23 was BamHI (GGATCC)-XhoI (CTCGAG). The plasmids and vectors were constructed by IBSBIO (Shanghai). Lipo6000 transfection reagent and plasmids were diluted with DMEM: 2.5 µl transfection reagent with 125 µl DMEM; 2.5 µg vectors with 125 µl DMEM. After remaining for 5 min, the two solutions were mixed gently and allowed to stand for another 20 min. Then, 250 µl of the mixture was added to each well, mixed gently and incubated at 37°C. Six hours later, the medium was changed, and the cells were cultured for 48 h.

### 2.8 MTS assay

PASMCs in 96-well plates with different treatments, 100 µl of 3-(4,5-dimethylthiazol-2-yl)-5- (3-carboxymethoxyphenyl)-2-(4-sulfophenyl)- 2H- tetrazolium (MTS) mixture (20 µl of MTS with 80 µl of DMEM) per well were incubated for 1 h. Then, the absorbance value of the plate was read at 490 nm wavelength (Wang et al., 2021; Zhang et al., 2021).

### 2.9 EdU assay

PASMCs were incubated with 50 µM 5-ethynyl-2′-deoxyuridine (EdU) for 2 h and then fixed with 4% paraformaldehyde. The cells were decolored using 2 mg/ml glycine, and then incubated with 0.5% Triton X-100 for 20 min. After the cells were washed, they were reacted with click-iT EdU mixtures for 30 min and then incubated with Hoechst 33342 for 30 min in the dark. The images were taken with a fluorescence microscope (Zhang et al., 2018; Zhang et al., 2021). The ratio of EdU positive cells to Hoechst positive cells was calculated.

### 2.10 Dual-luciferase^®^ reporter assay

The successfully constructed overexpression vectors were inoculated into 5 ml LB medium, and the plasmids were prepared by shaking (220 rpm) at a 37°C overnight, and the high purity plasmid extraction kit was used. Then, the reporter gene plasmids were transfected into HEK293 cells (DMEM 50 μl, gene overexpression plasmids (OE/NC) 450 ng, promoter reporter plasmids (Pro/NC) 75 ng, pRL-TK 25 ng, HG transgene reagent 1.5 μg), and the samples were collected. The samples were tested according to the instructions of the reporter gene detection kit (Dual-Luciferase^®^ Reporter Assay System, Promega, USA). Briefly, the lysate was centrifuged at 13,000 rpm for 5 min, and the supernatant was pooled. 20 μl samples were collected into a test tube, and then 20 μl of firefly luciferase assay reagent was added and mixed well to measure the relative light units (RLUs). Cell lysis buffer was used as a blank control well. Then, 20 μl of the prepared Renilla Luciferase Assay working solution was added to the tested sample, and the RLU was determined. The activation degree of the reporter gene was statistically analyzed. Pgl3-basic vector map and its vector information:


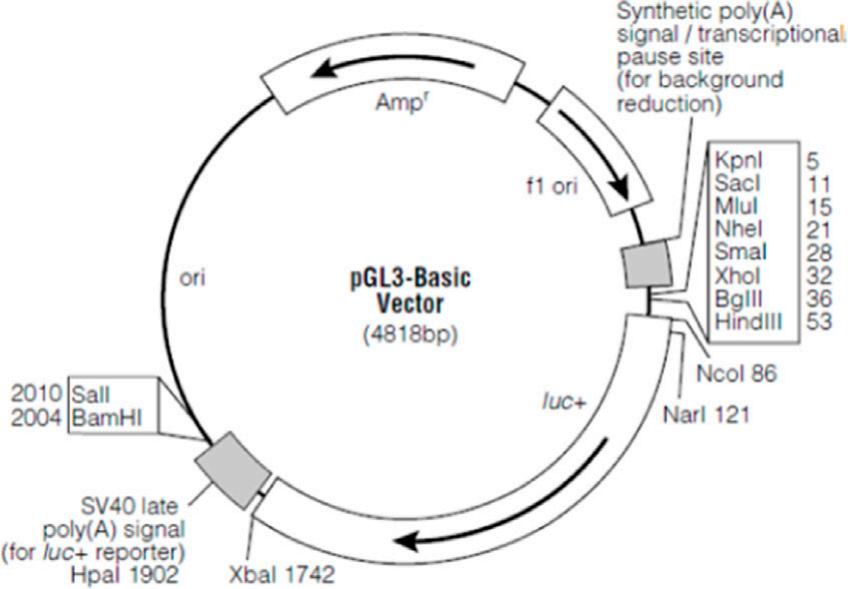

Pgl3-basic-Tex261 promoter WT: Cloning sites, KpnI (GGTACC)-XhoI (CTCGAG), Insert size, 612 bp;
Pgl3-basic-Tex261 promoter Mut: Cloning sites, KpnI (GGTACC)-XhoI (CTCGAG), Insert size, 604 bp;
pCDNA3.1-Hif1a: Cloning sites, BamHI (GGATCC)-XhoI (CTCGAG), Insert size, 2472 bp.

### 2.11 Real-time PCR

Total RNA from PAs and PASMCs was extracted by TRIzol, and then reverse transcribed into cDNA using a Roche Transcriptor First Strand cDNA Synthesis Kit (Roche, Germany). The fluorescence signal from SYBR Green combined with the newly synthesized double-stranded DNA was used for quantitative analysis (Roche, Germany). The primers used in this study are shown.

Tex261:sense, 5′-TTG​ATG​AAG​GAG​AAG​ACA​ACC​AGG​ATG-3′
antisens, 5′-CCA​TGC​AGC​CAG​GCG​ATG​AC-3′
HIF-1a:sense, 5′-TGA​GGC​TGT​CCG​ACT​GTG​AGT​AC-3′
antisens, 5′-CAC​CGC​AAC​TTG​CCA​CCA​CTG-3′
β-actin: sense, 5′-CCA​TCG​GCA​ATG​AGC​GGT​TCC-3′
antisense, 5′-CGT​GTT​GGC​GTA​GAG​GTC​CTT​G-3′

The specificity of the fluorescence signal was detected according to the melting curves. The data were obtained using a relative quantitative method (Zhang et al., 2018; Zhang et al., 2019).

### 2.12 Western blot

Total proteins were extracted from PAs of animal models and PASMCs with different treatments. RIPA (P0013B, Beyotime) contained PMSF (ST506, Beyotime) were added to lyse tissue or cells, and then centrifuged at 4°C, 14,000 rpm for 10 min. The supernatant was collected for Western blot experiments. 50 μg samples were isolated with a 10% SDS polyacrylamide gel and then transferred to PVDF membranes. After blocking with 5% skim milk for 2 h, the membranes were incubated with primary antibodies against Tex261 (1:500, Novus), Hif-1a, PCNA, Ndgr1, p-Akt, Akt (1:500, Beyotime) and Sec23a (1:5000, Abcam) at 4 °C overnight. After secondary antibodies were incubated for 2 h, the blots were reacted with enhanced chemiluminescence (ECL) reagent (Thermo, United States) for 5 min. Exposure detection was performed under a Biomolecular Imager. The relative optical density was analyzed, and β-actin was used as the control.

### 2.13 Intratracheal instillation of AVV6

#### 2.13.1 Prevention models

Rats were randomly divided into four groups: control + AAV6-GFP, control + AAV6-Tex261-GFP, Sugen5416/Hypoxia (Su/Hx)+AAV6-GFP, and Sugen5416/Hypoxia (Su/Hx)+AAV6-Tex261-GFP. AAV6 was intratracheally instilled first for 2 w and then subjected to hypoxia after Su5416 injection for 21 days to establish PAH models.

#### 2.13.2 Treatment models

Rats were randomly divided into three groups: control + AAV6-GFP, Sugen5416/Hypoxia (Su/Hx)+AAV6-GFP, and Sugen5416/Hypoxia (Su/Hx)+AAV6-Tex261-GFP. For establishment of the PAH model, first, the rats were injected with Su5416 and exposure to hypoxia for 21 days, and then instilled with AAV6 and rose for another 2 w in a normal environment.

### 2.14 Data analysis

Data are expressed as the mean ± SEM, and statistical analysis and mapping of the data were performed using GraphPad Prism 5. *T* tests were used for the comparison of the mean between the two groups, and one-way ANOVA was used for the comparison of the mean among the groups. *p* < 0.05 indicated a statistically significant difference.

## 3 Results

### 3.1 Tex261 is downregulated in hypoxic pulmonary arteries and pulmonary artery smooth muscle cells

**FIGURE 1:**
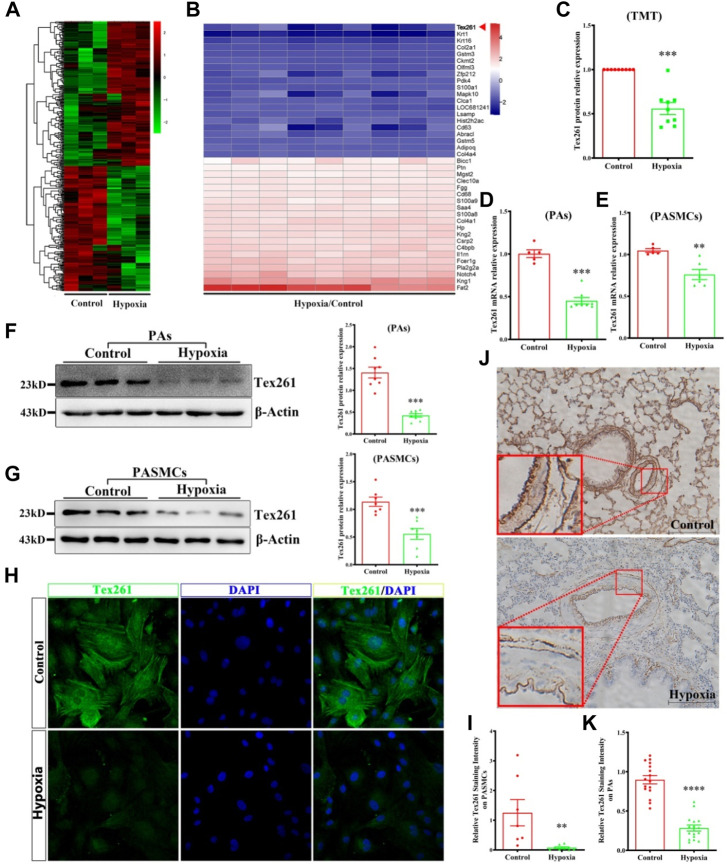
Tex261 is downregulated in hypoxic PAs and PASMCs. **(A)** Protein expression heatmap of hypoxia *versus* the control (*n* = 15): unsupervised hierarchical clustering analysis of the significantly dysregulated proteins. Red: upregulation; green: downregulation. **(B)** The heatmap shows the Top 20 of the significantly upregulated and downregulated proteins in the hypoxia group. **(C)** Tex261 protein relative expression chart in TMT data. **(D,E)** The mRNA levels of Tex261 in PAs and PASMCs were detected by real-time PCR (*n* = 5–8). **(F,G)** The protein expression of Tex261 in PAs and PASMCs was measured by Western blotting (*n* = 7–8). **(H,I)** The localization and expression of Tex261 in PASMCs were detected by immunofluorescence and observed under a ×20 microscope. **(J,K)** Tex261 mRNA was measured through ISH and observed under a ×20 microscope. The values are the mean ± SEM. *p < 0.05, **p < 0.01 and ***p < 0.001 vs. the control.

Based on TMT data, 811 differentially expressed proteins were obtained according to the criteria (fold-change >1.2, *p* < 0.05); 356 were upregulated and 455 were downregulated. The heatmap of the differentially expressed proteins is shown in Figure 1A. The top 20 upregulated and downregulated proteins are shown in Figure 1B. In this study, the Tex261 protein was selected because it was significantly reduced in hypoxic PAs (Figure 1C). To verify the mRNA and protein expression of Tex261 in both PAs and PASMCs after hypoxia, we used real-time PCR and Western blotting, respectively. As shown in Figures 1D–G, hypoxia indeed reduced the mRNA and protein expression levels of Tex261 in both PAs and PASMCs. Moreover, cell immunofluorescence showed that the Tex261 intensity was weakened in PASMCs after hypoxia and mainly existed in the cytoplasm, cytoskeleton and apparently stress fibers. (Figures 1H,I). Tex261 appears to be seen in the medial layers of vessels, suggesting its expression in smooth muscle, observed by ISH (Figures 1J,K). These results indicated that Tex261 was downregulated in both PAs and PASMCs under hypoxia.

### 3.2 Tex261 is negatively related to hypoxia and regulated by Hif-1α

**FIGURE 2:**
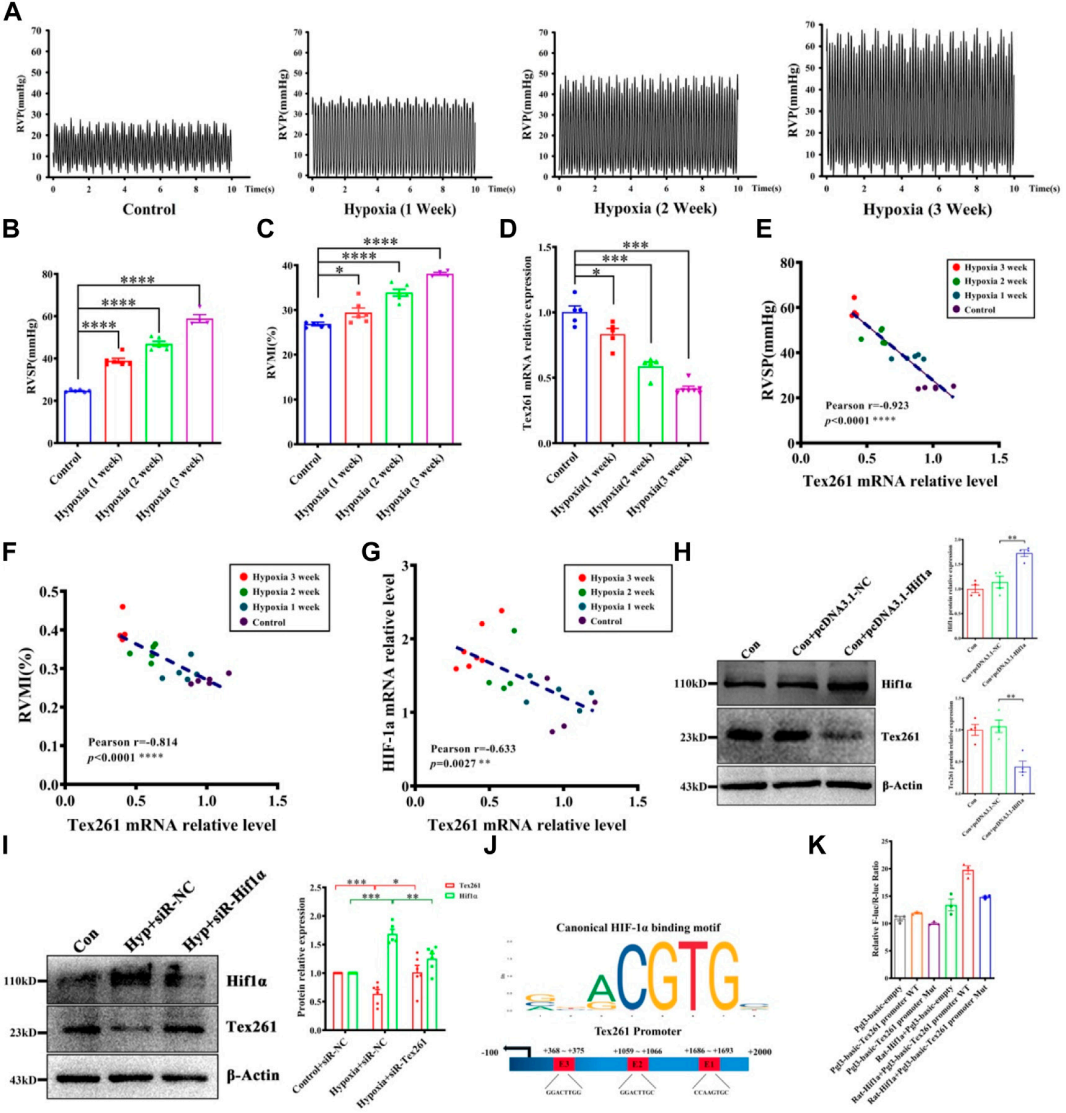
Tex261 is negatively related to hypoxia and regulated by Hif-1α. **(A)** Representative tracings of right ventricle pressure (PVP) recorded from different hypoxia time courses (0 W, 1 W, 2 W, 3 W). **(B,C)** The right ventricular systolic pressure (RVSP) and right ventricular mass index (RVMI) statistical graphs of different hypoxia time courses. (*n* = 4–6) **(D)** The mRNA levels of Tex261 in different hypoxia time courses (*n* = 5–7). **(E,F)** Correlation analysis between the mRNA levels of Tex261 and RVSP or RVMI. **(G)** Correlation analysis of the mRNA levels of Tex261 and Hif-1α. **(H)** Tex261 expression was detected after Hif-1α overexpression by Western blots (*n* = 4). **(I)** Tex261 expression was detected after Hif-1α interference by Western blots (*n* = 6). **(J)** Prediction of the binding sites between Hif-1α and Tex261. **(K)** Hif-1α binding with the promoter of Tex261 was identified by luciferase reporter gene assays (*n* = 3). The values are the mean ± SEM. *p < 0.05, ***p < 0.001 and ****p < 0.0001 vs. the control. **p < 0.01 vs. con + pcDNA3.1-NC. *p < 0.05, **p < 0.01 and ***p < 0.001 vs. the control + siR-NC or hypoxia + siR-NC.

To prove the relationship between Tex261 and PAH, we established animal models with different hypoxia durations (0 W, 1 W, 2 W, 3 W). As shown in Figures 2A–C, RVSP and RVMI gradually increased with prolonged hypoxia times. We also isolated PAs to detect the mRNA levels of Tex261. The results showed that the Tex261 mRNA levels decreased as the hypoxia time increased, as shown in Figure 2D. The correlations between Tex261 mRNAs and RVSP or RVMI were analyzed. We observed that both RVSP and RVMI had a negative correlation with Tex261 expression (Figures 2E,F). Furthermore, we found that the expression of Hif-1α and Tex261 had a negative correlation, as shown in Figure 2G. We examined whether Tex261 is regulated by Hif-1α. Overexpression of Hif-1α with vectors and Tex261 was detected in PASMCs. As shown in Figure 2H, elevated Hif-1α depressed Tex261 expression (Figure 2H). Moreover, Hif-1α was silenced in PASMCs with siRNAs, and Tex261 was detected in PASMCs after hypoxia. As shown in Figure 2I, Hif-1α knockdown reversed the hypoxia-induced Tex261 decrease. The JASPAR database (http://jaspar.genereg.net/) was used to predict the binding sites between Hif-1α and Tex261 (Figure 2J). The binding site was confirmed by luciferase reporter gene assay, as shown in Figure 2K. These results suggest that Tex261 is involved in the development of PAH and is regulated by Hif-1α under hypoxia.

### 3.3 Tex261 regulates the proliferation of pulmonary artery smooth muscle cells

**FIGURE 3:**
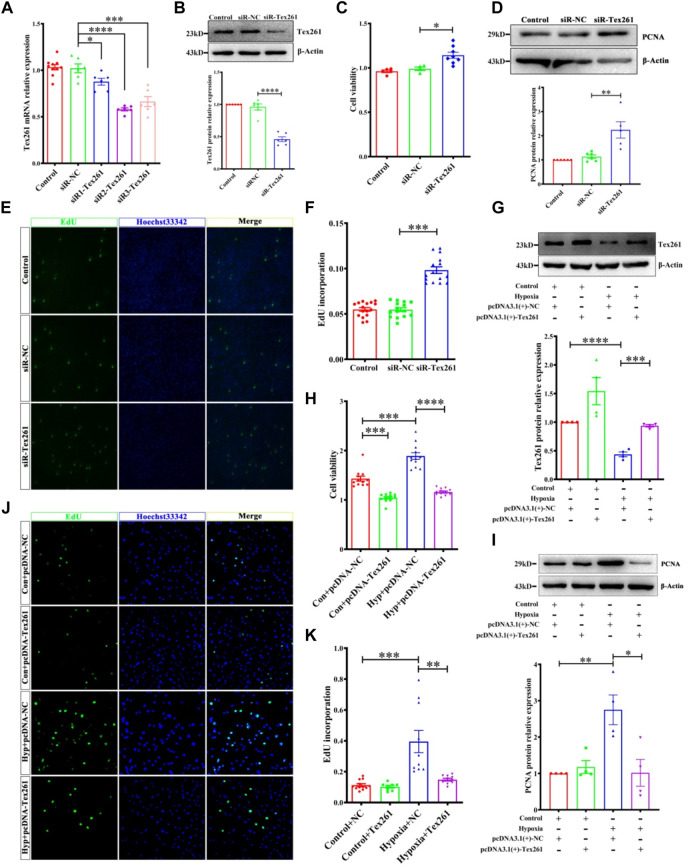
Tex261 regulates the proliferation of PASMCs. **(A,B)** Cell knockdown of Tex261 with siRNAs was verified in PASMCs (*n* = 6). **(C)** Cell viability was detected after transfection with siR-Tex261 or siR-NC in PASMCs by MTS assays (*n* = 4–8). **(D)** PCNA expression was measured after Tex261 silencing (n = 6). **(E,F)** The proportion of PASMC proliferation after Tex261 silencing was detected by EdU assays and observed by ×20 magnification (*n* = 6). **(G)** Western blot results showed Tex261 overexpression by transfection with plasmids in PASMCs (*n* = 4). **(H)** Cell viability was detected after Tex261 overexpression by MTS. **(I)** PCNA expression was measured after Tex261 overexpression (*n* = 4). **(J,K)** The proportion of PASMCs proliferating was detected by EdU assays and observed by ×20 microscope. Data are presented as the mean ± SEM. *p < 0.05, **p < 0.01 and ***p < 0.001 vs. siR-NC. ***p < 0.001 and ****p < 0.0001 vs. the control + pcDNA-NC or hypoxia + pcDNA-NC.

To observe the effect of Tex261 on the proliferation of PASMCs, we knocked down Tex261 expression, and the interference efficiency was verified, as shown in Figures 3A,B siR2-Tex261 has the most obvious interference efficiency, so it was used in subsequent experiments. Cell viability was detected by MTS assays. As shown in Figure 3C, silencing Tex261 enhanced the viability of PASMCs. PCNA expression was also significantly increased (Figure 3D). We also detected the cell proliferation rate after Tex261 knockdown through EdU incorporation assays. The results showed that the proportion of proliferating cells was significantly increased after Tex261 inhibition (Figures 3E,F). Correspondingly, cells overexpressed Tex261 vectors, as verified in Figure 3G. Figure 3H showed that Tex261 overexpression inhibited cell viability caused by hypoxia. Meanwhile, PCNA expression (Figure 3I) and cell proliferation (Figures 3J,K) were resumed after Tex261 overexpression. These results suggest that Tex261 regulates hypoxia-induced PASMC proliferation.

### 3.4 Sec23 is involved in Tex261-induced cell proliferation

**FIGURE 4:**
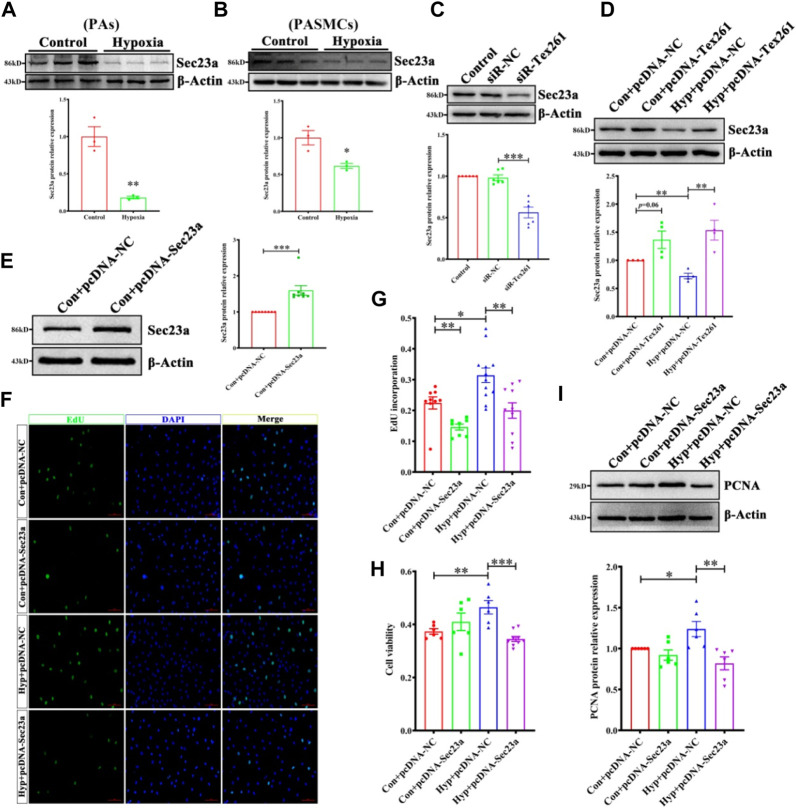
Sec23 is involved in Tex261-induced cell proliferation. **(A,B)** Sec23 expression was detected in hypoxic PAs and PASMCs (*n* = 3). **(C)** After Tex261 inhibition, Sec23 expression in PASMCs was detected by Western blots (*n* = 6). **(D)** After Tex261 overexpression, Sec23 was detected by Western blots (*n* = 4). **(E)** Western blot results showed that Sec23 increased after transfection with plasmids in PASMCs (*n* = 8). **(F,G)** The proportion of PASMCs proliferating was detected by EdU assays and observed by ×20 magnification (*n* = 6). **(H)** Cell viability was detected after Sec23 overexpression by MTS assays (*n* = 6). **(I)** PCNA expression was measured after Sec23 overexpression (*n* = 6). Data are presented as the mean ± SEM. *p < 0.05 and **p < 0.01 vs. the control. *p < 0.05, **p < 0.01, ***p < 0.001 vs. hypoxia + pcDNA.

Studies have shown that Tex261 is involved in the formation of COP II (Koyama et al., 2014). Through prediction, we found that Tex261 may interact with Sec23. Therefore, this study investigated the possible role of Sec23 in Tex261-induced cell proliferation. As shown in Figures 4A,B, Sec23 expression was decreased in both hypoxic PAs and PASMCs. After silencing of Tex261, Sec23 was decreased, while after Tex261 overexpression, Sec23 was increased in PASMCs (Figures 4C,D). The efficiency of Sec23 plasmid transfection is shown in Figure 4E. Overexpression of Sec23 inhibited hypoxia-enhanced cell viability and proliferation (Figures 4F–H), and also PCNA expression (Figure 4I), suggesting that Sec23 was involved in Tex261-induced PASMC proliferation.

### 3.5 Tex261 regulates Sec23 through Ndrg1 mediated akt pathways

**FIGURE 5:**
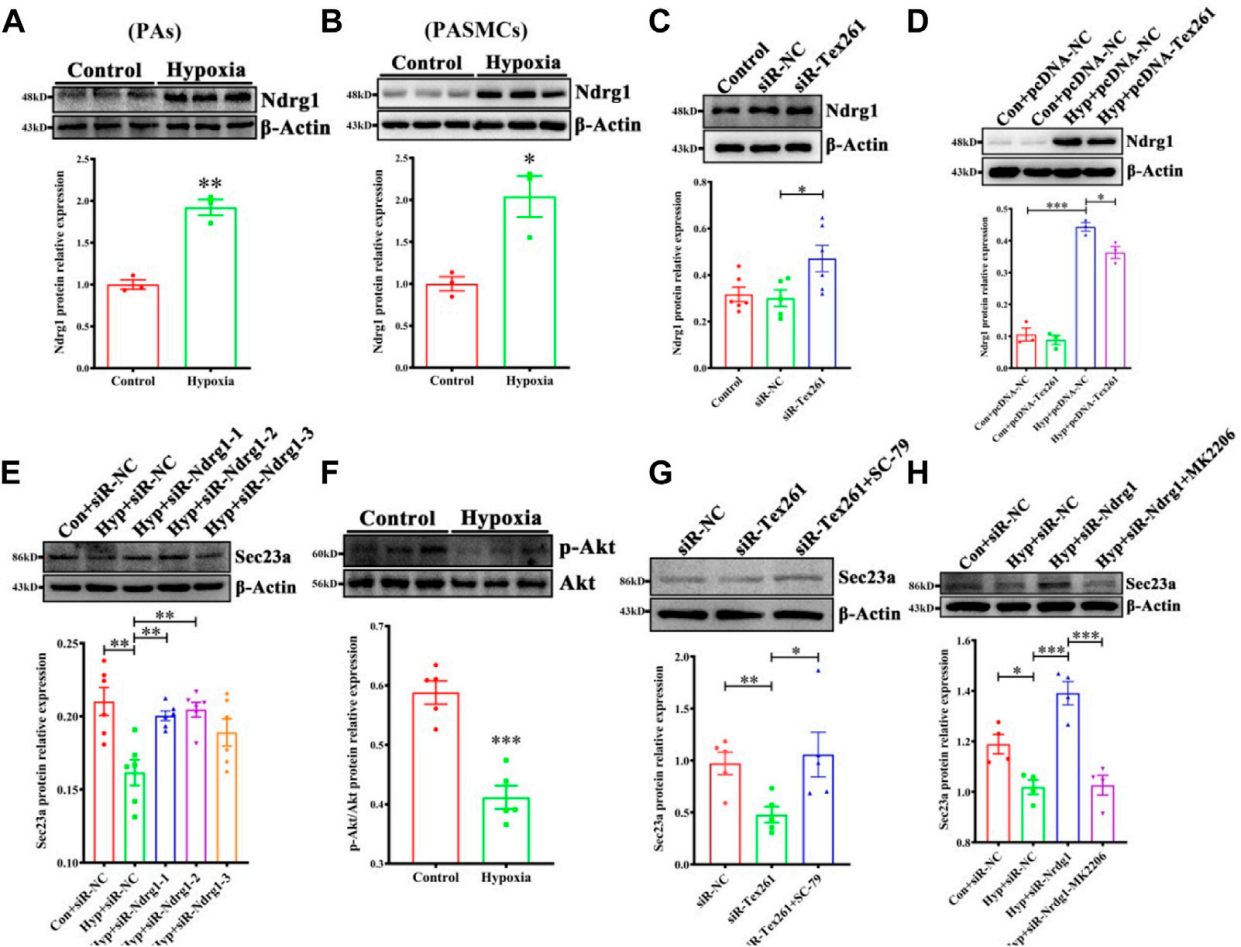
Tex261 regulates Sec23 through Ndrg1 mediated Akt pathways. **(A,B)** Ndrg1 expression was detected in hypoxic PAs and PASMCs (*n* = 3). **(C)** After Tex261 silencing, Ndrg1 expression was detected in PASMCs by Western blots (*n* = 6). **(D)** After Tex261 overexpression, Ndrg1 was measured (*n* = 4). **(E)** After Ndrg1 knockdown, Sec23 expression was examined (*n* = 6). **(F)** The Akt pathway was detected after PASMCs were exposed to hypoxia (*n* = 5). **(G)** Cells were pretreated with the Akt agonist SC-79 before Tex261 knockdown, and Sec23 was then measured (*n* = 5). **(H)** Cells were pretreated with the Akt blocker MK2206 before silencing Ndrg1, and Sec23 was then tested (*n* = 4). Data are presented as the mean ± SEM. *p < 0.05 and **p < 0.01 vs. control. *p < 0.05 and ***p < 0.001 vs. con + pcDNA3.1-NC. *p < 0.05, **p < 0.01 and ***p < 0.001 vs. the control + siR-NC or hypoxia + siR-NC.

Ndrg1 may be a controversial factor in cancers (Bandyopadhyay et al., 2004; Wang et al., 2019). Whether Ndrg1 is regulated by Tex261 is unclear. First, we detected Ndrg1 expression after hypoxia. The data showed that hypoxia increased Ndrg1 in both PAs and PASMCs (Figures 5A,B). Moreover, Tex261 knockdown enhanced Ndrg1 expression, while Tex261 overexpression suppressed it (Figures 5C,D). When Ndrg1 was silenced, Sec23 expression increased, as shown in Figure 5E. Furthermore, Ndrg1 may regulate Akt pathway activity. In our study, hypoxia inactivated the Akt pathway (Figure 5F). Cells were pretreated with the Akt pathway agonist SC-97, and then silenced Tex261, Sec23 expression was resumed (Figure 5G). Cells pretreated with the Akt inhibitor MK2206 before Ndrg1 knockdown reversed Sec23 expression, as shown in Figure 5H. These results indicated that Ndrg1 medicated Akt is involved in Tex261 regulation of Sec23.

### 3.6 Preventive effect of Tex261 on rats with pulmonary artery hypertension

**FIGURE 6:**
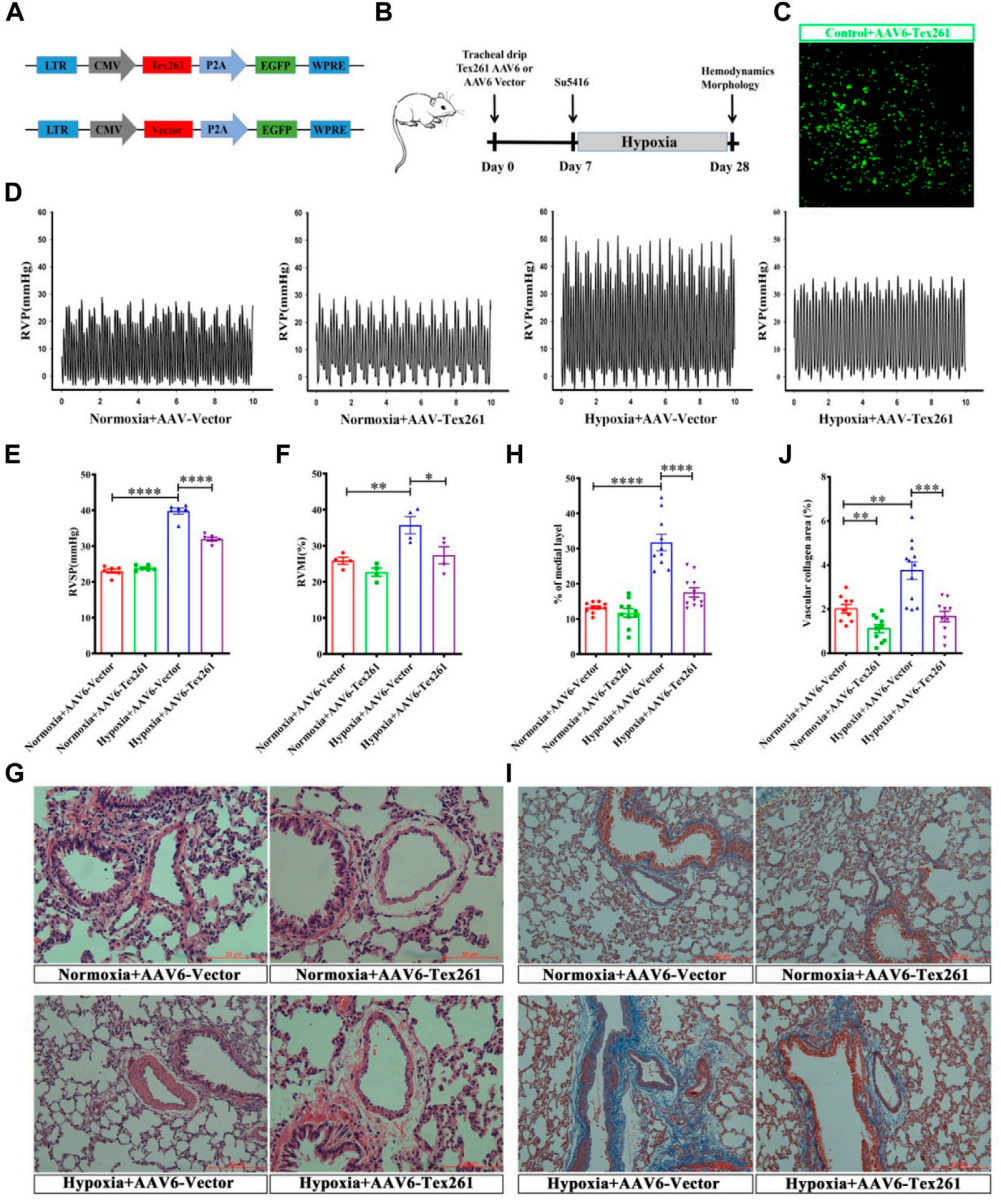
The preventative effects of Tex261 on rats with PAH. **(A)** Schematic diagram of the structure of the AAV6-Tex261-GFP shuttle. **(B)** Animal treatment protocols for the preventative effects of Tex261. After transfection of AAV6, hemodynamics and morphology were recorded after Su5416 administration at the beginning of hypoxia. **(C)** Verification of Tex261 transfection in lung tissues with immunofluorescence and observation by 10x confocal microscopy. **(D–F)** Representative tracings of RVP, RVSP and RVMI recorded from the control and hypoxic rats treated with AAV6-GFP and AAV6-Tex261-GFP, respectively. **(G,H)** PAR was observed through HE staining and observed by a ×20 microscope. The PAR was calculated according to the average of the four diagonal thicknesses divided by the diameter of the vessel. **(I,J)** Vascular fibrosis was detected with Masson staining and observed by ×20 microscopy. Data are presented as the mean ± SEM. *n* = 6, *p < 0.05, **p < 0.01 and ***p < 0.001 vs. hypoxia + AAV6-GFP.

Tex261 was overexpressed by adeno-associated virus 6 (AAV6), and the construction mode is shown in Figure 6A. Two weeks after tracheal infusion of AAV6-Tex261-GFP or AAV6-GFP, rats were fed in a normal or hypoxic environment for another 3 weeks after Su5416 injection, to observe the preventive effect of Tex261 expression on PAH rats. The diagram of the specific animal experiment scheme is shown in Figure 6B. Lung infection with AAV6-GFP was observed through a frozen section scan. The images showed that AAV6 had been integrated into the lung tissues and expressed (Figure 6C). Then, RVSP was monitored, and RVMI was calculated. We found that the RVSP and RVMI of Sugen5416/hypoxic rats were significantly relieved after injection of the Tex261 virus (Figures 6D–F). Pulmonary vessel walls were thinned with AAV-Tex261 (Figures 6G,H). Artery fibrosis also resumed after the Tex261 increase (Figures 6I,J). These results indicated that Tex261 could prevent PAH to some extent.

### 3.7 The therapeutic effect of Tex261 on rats with pulmonary artery hypertension

**FIGURE 7:**
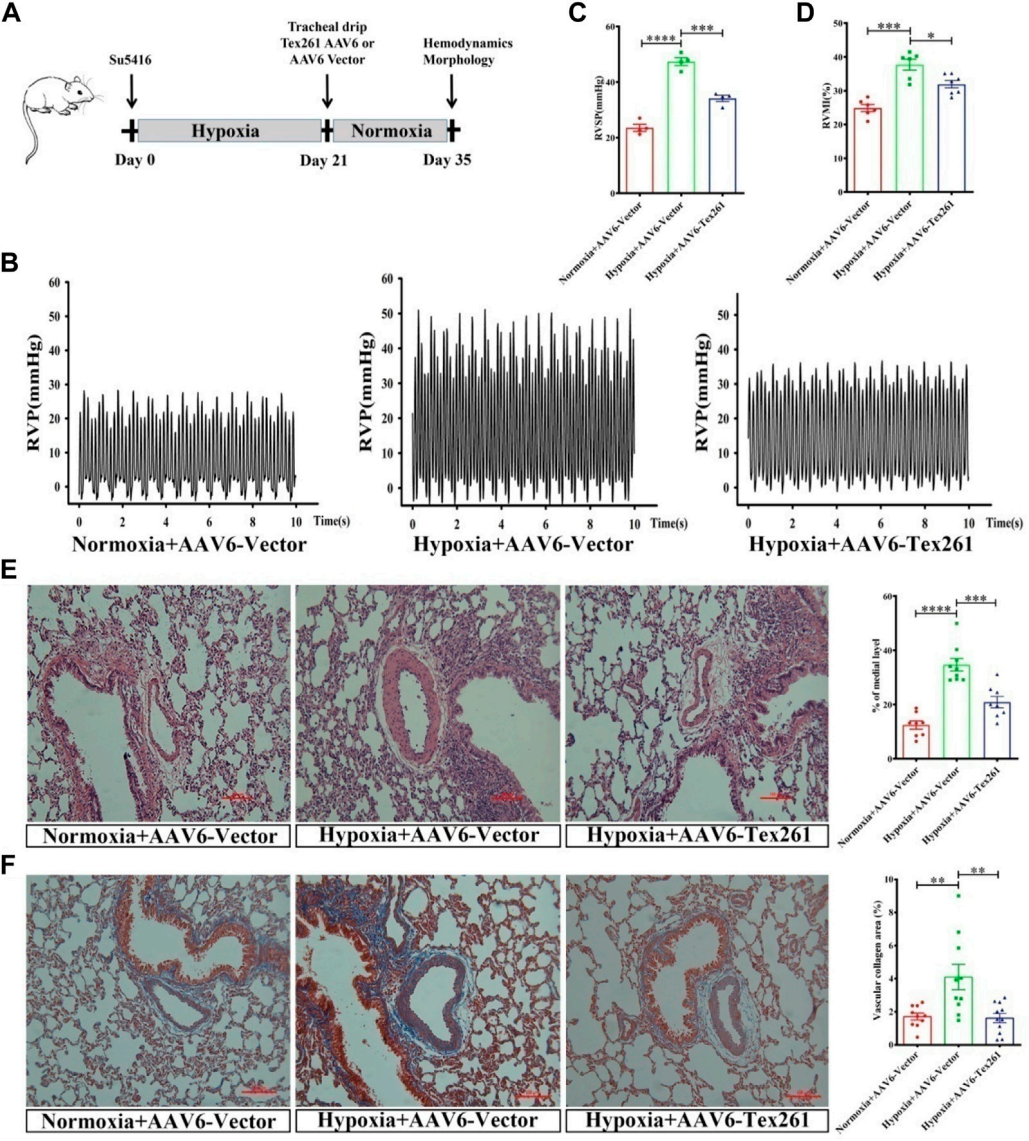
The therapeutic effect of Tex261 on rats with PAH. **(A)** Animal treatment protocols for the therapeutic effect of Tex261. After administration of Su5416, rats were exposed to hypoxia for 3 W to establish a PAH model, and then, AAV6 was injected and the animals were fed for 2 W. Hemodynamics and morphology were recorded. **(B–D)** Representative tracings of RVP, RVSP and RVMI recorded from the control + AAV6-GFP, hypoxia + AAV6-GFP, and hypoxia + AAV6-Tex261-GFP mice. **(E)** PAR was observed by HE staining and observed by a ×20 microscopy. The PAR was calculated according to the average of the four diagonal thicknesses divided by the diameter of the vessel. **(F)** Vascular fibrosis was detected by Masson staining and observed by ×20 microscopy. Data are presented as the mean ± SEM. *n* = 4–6, *p < 0.05, **p < 0.01 and ***p < 0.001 vs. hypoxia + AAV6-GFP.

Rats were injected with Su5416 and then subjected to hypoxia for 3 W to establish PAH models, and then AVV6 w as injected and breaded for another 2 W, to observe the therapeutic effect of Tex261 expression on PAH rats. The diagram of the specific animal experiment scheme is shown in Figure 7A. Sugen5416/Hypoxia significantly increased RVSP and RVMI, while treatment with AAV6-Tex261 rescued them (Figures 7B–D). HE and Masson staining results showed that Tex261 overexpression alleviated PAR and fibrosis (Figures 7E,F). The results suggested that Tex261 may have a therapeutic effect on rats with PAH.

## 4 Discussion

PAH is an extremely malignant cardiovascular disease with progressive PAR or occlusion, and eventually leads to death (Southgate et al., 2020). Currently, the drugs for PAH treatment mainly aim to reduce the symptoms of patients and improve the quality of life of patients (Coons et al., 2019; Galiè et al., 2019). However, they cannot reverse the progression of the disease. Therefore, there is an urgent need to search for new targets for drugs. This study indicated that Tex261 was downregulated in both rats with PAH and PASMCs. Hif-1α regulated Tex261 expression under hypoxia. Tex261 inhibited PASMC proliferation. Overexpression of Tex261 using AVV6 alleviated the increase in RVP and PAR caused by Sugen5416/hypoxia, which may have certain preventive and therapeutic effects on rats with PAH.

Tex261 is mainly involved in the formation of COP II. COP II is known to mediate the transport of proteins from the endoplasmic reticulum to the Golgi apparatus (Mossessova et al., 2003). Studies have found that Tex261 might regulate excitotoxic cell death induced by NMDA receptor activation (Miller et al., 2003). Tex261, as a target of miR-28-5p in prostate cancer, affects cell proliferation, survival and apoptosis (Rizzo et al., 2017). Moreover, the Tex261 family contains many members, among which Tex9 is believed to cooperate with eIF3b to promote proliferation and inhibit apoptosis of esophageal squamous cells and carcinoma occurrence by activating the AKT signaling pathway (Burg et al., 2008). In this study, we found that Tex261 was significantly reduced in TMT sequencing data. To prove that there was no a concomitant change in PAH, we established different time points in hypoxic rats and detected the possible roles of Tex261 in the development of PAH. The results showed that Tex261 gradually decreased with prolonged hypoxia and increased PVP, suggesting that Tex261 may be a factor in the onset of PAH. Moreover, decreased Tex261 could regulate PASMC proliferation and participate in PVR. Proliferating smooth muscle cells could migrate to intima and participate in intima remodeling as reported. We are also checking the effects of Tex261 on the migration, phenotype and apoptosis of PASMCs, which is a follow-up work of this study and are thinking to include those data in their next paper. Furthermore, we also found that overexpression of Tex261 alleviated fibrosis, and the possible mechanism was that Tex261 has a certain regulatory effect on adventitia as we observed it was decreased after hypoxia in Figure 1J.

Hif-1α is closely related to PAH, and may be a driving factor upstream of PAH (Kojima et al., 2019; Luo et al., 2019). How hypoxia inhibits Tex261 expression has not yet been reported. We found that the expression of Hif-1α and Tex261 was negatively correlated under hypoxia. The JASPAR database was used to predict the binding sequence sites of the Hif-1α and Tex261 promoters. The results also verified that Hif-1α regulated the promoter activity of Tex261 and suppressed Tex261 expression.

Tex261 is closely related to COP II vesicle transport, and Sec23 is one of the important components of COP II. Reports have shown that the abnormal expression and mutation of Sec23 cause various diseases (Boyadjiev et al., 2006; Lang et al., 2006; Fromme et al., 2007). Sec23 is a direct target of miR-200c, which mediated the secretion of metastasis inhibitory proteins such as IGFBP4. Sec23 down-regulation was closely related to proliferation and metastasis (Long et al., 2013). Overexpression of miR-21 inhibited Sec23 and promoted the proliferation, migration and invasion of DLD-1 cells. The same results were obtained by knocking out Sec23 in DLD-1 cells (Yang et al., 2013). In LNCaP and DU145 CaP cells, Sec23 overexpression suppressed cell growth, while Sec23 inhibition promoted cell proliferation (Jones et al., 2003). We observed that Sec23 was reduced after hypoxia and participated in the regulation of PASMC proliferation and PAR.

Ndrg1 is a component of ER stress because it is very sensitive to the redox state of cells and intracellular calcium concentration (Okuda et al., 2005). Ndrg1 is involved in the induction of ER-induced partners, which may itself be a companion protein or a target of these partners (Kokame et al., 1998; Lan Chun Tu et al., 2007). Ndrg1 belongs to a new protein family that does not contain protein motifs with known functions (Zhou et al., 2001). Studies have shown that Ndrg1-interacting proteins contain Sec23a through LC‒MS/MS^31^, but their role has not been reported in PAH and other diseases. Therefore, we tested whether Sec23 was regulated by Tex261 and mediated by Ndrg1. We found hypoxia increased Ndrg1 and was regulated by Tex261, while Ndrg1 knockdown restored Sec23 expression. The results seemed contradictory with others (Lan Chun Tu et al., 2007). Reports have shown that Ndrg1 is increased in liver cancer, kidney cancer and other solid cancers, but it is decreased in colon, nervous system and other tumors (Melotte et al., 2010). Sec23 was reduced in PASMCs after hypoxia, so we speculated that Ndrg1 may not directly bind to Sec23 in PASMCs. This molecular may regulate Sec23 in other ways. To explore this problem, we focused on the Akt pathways because they play an important role in regulating cell functions. Studies have reported that increased Ndrg1 suppresses angiogenesis *via* the PI3K/Akt pathways in human placental cells (Dai et al., 2020). Ndrg1 suppressed EMT through activation of the Wnt/β-catenin signaling pathway (Chen et al., 2018). Ndrg1 was shown to modulate the Wnt-β-catenin pathway by inhibiting the nuclear translocation of β-catenin (Jin et al., 2014). Our results showed that hypoxia induced Akt inactivation, but treatment with Akt agonists or blockers reversed the change in Sec23 after Ndrg1 interference, indicating that Ndrg1 regulates Sec23 through Akt pathways. Moreover, Akt pathways were reported to play a role in PAH (de Jesus Perez et al., 2014; Li et al., 2020), and our results also showed that the pathways were involved in PASMC proliferation after hypoxia. Furthermore, we observed that Ndrg1 was enhanced and mainly expressed in the cytoplasm, while Sec23 was weakened, mainly in the nucleus after hypoxia stimulation through immunofluorescence, indicating that there were differences in expression in time and space between the two proteins. This finding may explain why there was no direct combination of Ndrg1 and Sec23 but indirect regulation of Sec23 by Ndrg1 through the Akt pathway in PASMCs.

## 5 Conclusion

Hypoxia inhibits Tex261 expression through Hif-1α, and lowered Tex261 promotes PASMC proliferation and PAH development through Ndrg1-Akt-induced Sec23 downregulation. Tex261 elevation alleviates PAH in rats. This study provides a new theoretical basis for elucidating the pathogenesis of PAH and potential treatment and prevention targets for PAH.

## Data Availability

The original contributions presented in the study are included in the article/supplementary material, further inquiries can be directed to the corresponding authors.
